# Adipose-derived stem cells enhance the tumorigenic potential of pre-malignant breast epithelial cells through paracrine activation of PI3K–AKT pathway

**DOI:** 10.1007/s12282-025-01686-7

**Published:** 2025-02-28

**Authors:** Qifeng Wu, Jinguang He, Tanja Herrler, Baofu Yu, Qimin Zhou, Danning Zheng, Xiaoxue Chen, Yangxuanyu Yan, Chuanchang Dai, Kai Liu, Gangming Zou, Shengfang Ge, Yunbo Qiao, Qingfeng Li, Jiao Wei

**Affiliations:** 1 Department of Plastic and Reconstructive Surgery, Shanghai Ninth People’s Hospital, Shanghai JiaoTong University School of Medicine, Shanghai, 200125 China; 2 Berufsgenossenschaftliche Unfallklinik Murnau, Murnau, Germany; 3 Gannan Medical University, Ganzhou City, Jiangxi China; 4 Department of Ophthalmology, Shanghai Key Laboratory of Orbital Diseases and Ocular Oncology, Shanghai Ninth People’s Hospital, Shanghai, China; 5 Shanghai Institute of Precision Medicine, Shanghai, 200125 China

**Keywords:** Breast cancer, Tumorigenesis, Adipose-derived stem cells, MCF-10AT cells, PI3K–AKT pathway

## Abstract

**Background:**

Adipose-derived stem cells (ADSCs)-assisted fat grafting has emerged as a widely used procedure for breast reconstruction post mastectomy and for aesthetic augmentation. Given the limited cases of breast cancer following grafting, the oncological safety of this procedure remains controversial.

**Methods:**

The effects of ADSCs on the oncogenic features of premalignant MCF-10AT cells were investigated using co-culture and xenograft models. We further evaluated the malignancy-promoting effect of ADSCs in a 7,12-Dimethylbenz(a)anthracene (DMBA)-induced breast cancer model. RNA-sequencing was performed on ADSCs, MCF-10AT cells, and ADSC-co-cultured MCF-10AT cells. Protein changes in ADSC/MCF-10AT co-culture medium and MCF-10AT cells were determined by proteomic analysis. Pathway inhibitors were used to investigate signaling pathways involved in the ADSC-induced oncogenic changes of MCF-10AT cells.

**Results:**

We found that ADSCs promoted the proliferation and migration of MCF-10AT cells, and co-injection of ADSCs increased the tumor incidence of MCF-10AT cells from 29% to 58% in nude mice. Additionally, grafted ADSCs significantly enhanced tumor incidence, growth, and distant metastasis in the DMBA-induced rats, while it could not induce tumorigenesis in normal breast tissues. Combined RNA-sequencing and proteomic analysis demonstrated that the paracrine factors secreted by ADSCs robustly activated the oncogenic PI3K–AKT signaling in MCF-10AT cells. We also revealed the auto-activated TGF-beta and Wnt pathways in co-cultured MCF-10AT cells, which may be synergistic in tumor formation and progression. As expected, blocking these pathways, especially the PI3K–AKT pathway, strongly diminished the promoting effects of ADSCs, suggesting their potential as therapeutic targets for ADSC grafting-associated breast tumors.

**Conclusions:**

Our data illustrated the synergistic effect between ADSC paracrine factors and MCF-10AT auto-activated pathways in the carcinogenesis of MCF-10AT cells through activation of the oncogenic PI3K–AKT pathway. Based on these findings, we strongly recommend pre-operative examinations for breast cancer risk factors before ADSC-associated transplantation.

**Supplementary Information:**

The online version contains supplementary material available at 10.1007/s12282-025-01686-7.

## Introduction

Fat grafting for breast reconstruction after mastectomy and aesthetic augmentation has increased in popularity, partly due to recent reports of silicone breast implant-associated anaplastic large cell lymphoma [1, 2]. Adipose-derived stem cells (ADSCs), crucial cellular components of adipose tissue (approximately 1.0 × 10^5^ ADSCs per gram) [3], contribute to angiogenesis and the long-term survival of the grafted fat [4]. The addition of ADSCs to fat tissue, known as cell-assisted lipotransfer (CAL), has become widely used in clinical practice to enhance graft retention [5, 6]. Despite its clinical benefits, the oncological safety of ADSCs remains controversial [7]. Previous studies have primarily focused on the interactions between ADSCs and breast cancer cells, revealing the potential for ADSCs to enhance tumor growth, progression, and metastasis both in vitro and in vivo [8]. For example, ADSC-secreted interleukin-6 (IL-6) can induce epithelial–mesenchymal transition (EMT) and promote invasion in both normal and primary breast tumor epithelial cells [9]. Adipsin, an adipokine primarily secreted by ADSCs, has been found to enhance cancer stem cell (CSC) properties in co-cultured breast cancer cells [10]. Additionally, tumor-secreted factors can modify ADSCs, leading to extracellular matrix (ECM) remodeling and further tumor progression [11]. These findings suggest that ADSCs may interact with the tumor microenvironment and exhibit tumor-promoting effects through paracrine mechanisms or direct stromal–tumor interactions with malignant cells.

However, some studies suggest that these oncogenic effects may be attenuated when ADSCs are integrated within autologous fat tissue [12, 13]. Although several preclinical and clinical studies have emphasized the oncological safety of CAL [14, 15], a few reported cases of breast carcinoma recurrence or new tumors following grafting [16] highlight potential oncogenic risks associated with ADSCs. Thus, we hypothesize that transplanted ADSCs may act as a promoting factor for tumor formation in specific genetic or non-genetic contexts, rather than an initiating factor for healthy epithelium.

To fill the gap in existing studies that predominantly focus on fully malignant breast cancer cells, we investigated the effects of human ADSCs on the oncogenic features of the premalignant breast cell line MCF-10AT through direct or transwell-mediated co-culture. In addition, the tumor-promoting effects of ADSCs were validated in vivo using xenograft and 7,12-Dimethylbenz(a)anthracene (DMBA)-induced mammary cancer models. Moreover, integrative RNA sequencing and proteomic analyses uncovered activation of the oncogenic PI3K–AKT signaling pathway by ADSC-secreted paracrine factors. These findings underscore the oncogenic potential of ADSCs in mammary primary tumorigenesis and highlight the importance of assessing the cancer risk background of CAL recipients before breast reconstruction or aesthetic surgery.

## Materials and methods

### Isolation, culture, and characterization of human and rat ADSCs

Human adipose-derived stem cells (ADSCs) were isolated from the abdominal or thigh liposuction samples of healthy female donors (age: 28 ± 6.1 years old; BMI: 20 ± 2.1 kg/m^2^; *n* = 6). Similarly, rat ADSCs (rADSCs) were derived from the inguinal fat pads of 8-week-old female Sprague–Dawley (SD) rats. The procedures for the isolation, culture, and flow cytometric characterization of ADSCs were performed as we previously described [17]. Cells from passages 2 to 4 were used in the subsequent experiments. The use of adipose tissue from liposuction was approved by patients and the informed consent was obtained.

### Characterization and culture of MCF-10AT and SHZ-88 cell lines

The MCF-10AT premalignant cell line was kindly provided by Professor Hongfeng Zhao (Mammary Gland Department, LongHua Hospital Shanghai University of TCM) from Barbara Ann Karmanos Cancer Institute (Michigan Cancer Foundation, USA). The rat breast cancer cell line SHZ-88 was purchased from the Chinese Academy of Sciences (Shanghai, China). MCF-10AT and SHZ-88 cells were cultured in high glucose DMEM (Servicebio, China) supplemented with 10% FBS (Gibco, USA) and 1% Penicillin–Streptomycin (Gibco, USA). All cells were maintained in a 37 °C incubator under a 5% CO_2_ atmosphere.

### Cell cycle analysis

ADSCs and MCF-10AT cells were also cultured in the transwell system for cell cycle analysis. After three days of culture, MCF-10AT cells were treated with 0.25% trypsin–EDTA (Gibco, USA), washed three times with PBS buffer, and fixed with 70% ethanol at 4 °C overnight. Fixed cells were then stained with 500 µl PI/RNase Staining solution (F10797, ThermoFisher, USA) for 30 min at room temperature. Flow cytometry was used to detect the red fluorescence at a 488 nm excitation wavelength (NovoCyte Advanteon, Agilent, USA). The cell cycle was analyzed and visualized using FlowJo (Version 10.8.1) software.

### Cell tracking of MCF-10AT cells in nude mice

In vivo xenograft experiments were conducted to track MCF-10AT cells in nude mice. Cells were implanted into the mammary fat pad of 8-week-old female athymic nude mice, which were divided into two groups (*n* = 30 per group): one group with GFP-fLuc MCF-10AT cells co-injected with ADSCs, and the other with only GFP-fLuc MCF-10AT cells. Specifically, 5.0 × 10^6^ cells suspended in 200 µl of culture medium were injected using a 25-gauge needle into sedated mice. Initial tumor formation in nude mice was noted when tumors reached a diameter of 3 mm, with detailed records of tumor characteristics including location, size, texture, boundaries, and signs of ulceration. At 6, 12, and 20 weeks, mice were euthanized to measure tumor volumes, and anatomical dissections were performed. Removed tumors were fixed in 4% formalin for further histological analysis. Tumor incidence was calculated using the entire cohort of animals, and tumor characteristics were evaluated by averaging data from all tumors collected at each corresponding time point.

### In vivo tumor growth and metastasis analyses in the DMBA-induced rat breast cancer model

8-week-old female SD rats were randomized into four groups (40 rats per group). The three experimental groups were treated with a single gavage of DMBA (10 mg/100 g, dissolved in sesame oil; Sigma-Aldrich, USA). One week before DMBA gavage, the experimental rats received one of the following subcutaneous injections into the second mammary gland area: 5.0 × 10^6^ rat ADSCs (rADSCs), 5.0 × 10^6^ SHZ-88 cells (the positive control group), or 0.5 ml of ADSC culture medium (CM). Meanwhile, the healthy control group received only the rADSCs injections and was not treated with DMBA. These injections were repeated three times at 21-day intervals. Tumor growth was recorded as described above. After intragastric administration of DMBA, animals were randomly sacrificed at different time points. Tumors and distant organ metastasis lesions in the lungs, spleen, liver, stomach, intestine, kidneys, uterus, and skeleton were detected with micro-PET-CT and macroscopically examined after fixation with 4% formalin. The metastasis index for each rat was calculated as the ratio of the number of tumor metastases divided by primary tumor weight (in grams). Tumor incidence was calculated using the entire cohort of animals, and tumor characteristics were evaluated by averaging data from all tumors collected at each corresponding time point.

### Pathway inhibitor assays

The roles of PI3K–AKT, TGF-beta, and Wnt pathways in the migration and proliferation of MCF-10AT cells co-cultured with ADSCs were assessed using pathway-targeted inhibitors: 0.5 μM BEZ235 for PI3K, 10 μM SB431542 for TGF-beta, and 20 μM IWR-1-endo for Wnt (all from Selleck, China). Cell migration was evaluated in the transwell system, and proliferation was measured under both transwell and direct co-culture conditions.

Other key methods and materials used in this study are detailed in the Supplementary Information.

## Results

### Human ADSCs promote the proliferation and migration of MCF-10AT cells

**Fig. 1:**
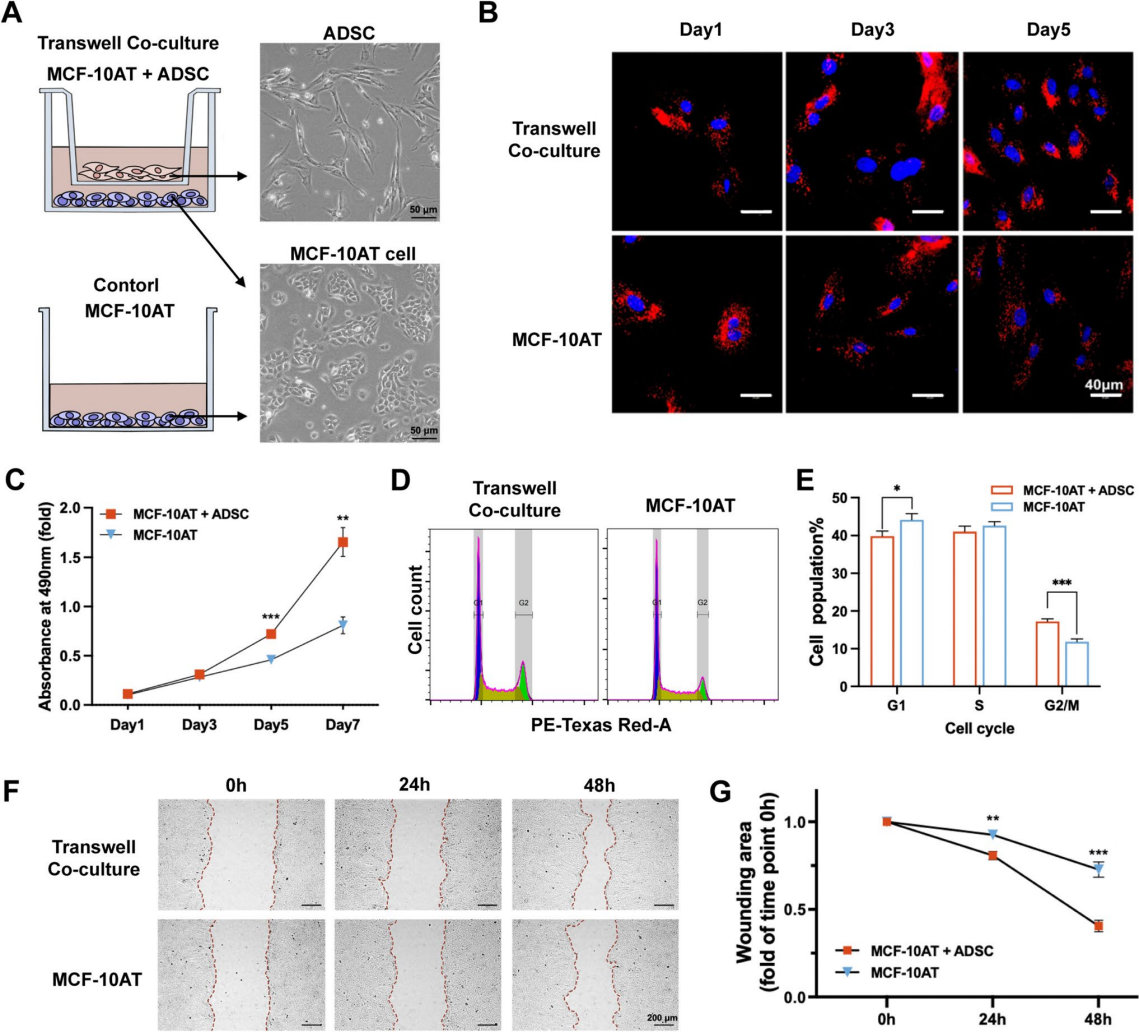
Human ADSCs enhance proliferation and migration of MCF-10AT cells in vitro*.* **A** Schematic of MCF-10AT cells cultured alone or in transwell co-culture with ADSCs for subsequent proliferation, cell cycle, and migration analyses. **B** Proliferation of ADSC-co-cultured or mock Dil-labeled MCF-10AT cells, stained with DAPI and visualized by confocal laser scanning microscopy. **C** MTT cell growth assay for MCF-10AT cells cultured alone or co-cultured with ADSCs. Statistical analysis of absorption at 490 nm is presented. **D**, **E** Flow cytometry cell cycle analysis showing the percentage of cells in different phases when cultured alone or with ADSCs. **F**, **G** Transwell scratch wound healing assay performed for MCF-10AT cells alone or with ADSC. Data in (**C**, **E** & **G**) are shown as means ± SD of biological replicates (*n* = 3), *p < 0.5, **p < 0.01, ***p < 0.001

We first analyzed the impact of ADSCs on the proliferation, cell cycle progression, and migration of MCF-10AT cells using a transwell co-culture system (Fig. 1A). For enhanced visualization, MCF-10AT cells were stained with 1,1′-Dioctadecyl-3,3,3′,3′-Tetramethylindocarbocyanine (DiI) and seeded either alone or with ADSCs in transwell chambers. Confocal laser scanning microscopy and MTT assays showed a significant increase in the proliferation rate of co-cultured MCF-10AT cells (Fig. 1B, C). Consistently, cell cycle analysis using propidium iodide (PI) staining followed by flow cytometry demonstrated a higher percentage of G2/M-phase cells in co-cultured MCF-10AT cells (17%) compared to controls (12%) (Fig. 1D, E). In addition, co-cultured MCF-10AT cells exhibited approximately a two-fold increase in cell migration, as quantified by the scratch wound healing assay compared to the control group (Fig. 1F, G) (detailed methods in Supplementary Information). These data suggest that co-culture with human ADSC promotes the proliferation and migration of MCF-10AT cells, possibly through a paracrine pathway.

### Human ADSCs enhance the tumorigenic potential of MCF-10AT cells

**Fig. 2:**
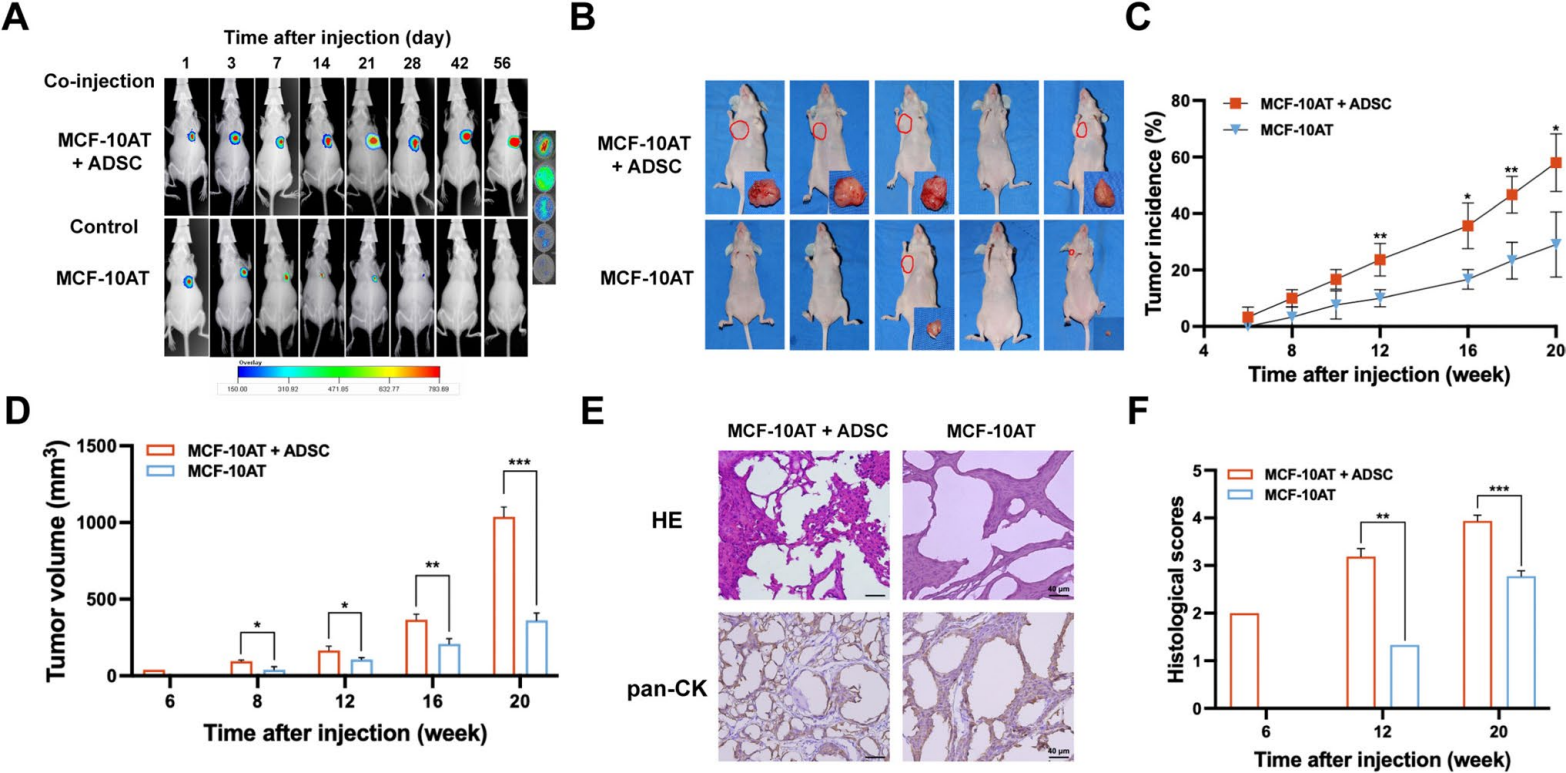
Human ADSCs promote tumorigenicity of MCF-10AT cells in nude mice. **A** Quantification of luminescence intensity of nude mice injected with GFP-fLuc MCF-10AT alone or co-injected with ADSCs, measured using the IVIS system. **B** Representative tumors from nude mice injected with MCF-10AT cells alone or co-injected with ADSCs at 20 weeks were presented. **C**, **D** Statistical analyses of tumor incidence (**C**) and tumor volume (**D**) in MCF-10AT only and ADSC co-injected groups from 6 to 20 weeks. **E** Histological features of tumors in ADSC co-injected group and MCF-10AT injected nude mice, demonstrated by Hematoxylin and Eosin (HE) staining and immunohistochemical (IHC) staining with human pan-cytokeratin (pan-CK). **F** The histological grading scores for breast lesions evaluated at 8, 12, and 20 weeks after MCF-10AT injection with or without ADSCs. Data in (**C**, **D**, **F**) are shown as means ± SD of biological replicates (*n* = 3–9/group at each time point), *p < 0.5, **p < 0.01, ***p < 0.001

MCF-10AT cells were derived from benign proliferative breast tissue and characterized by the expression of activated T24 H-ras and moderate tumor-forming abilities [18]. We then assessed whether human ADSCs could promote tumorigenesis of the premalignant MCF-10AT cells in athymic nude mice (Supplementary Fig. 1A). To track the cells in vivo, MCF-10AT cells were labeled with a lentivirus containing the firefly luciferase (fLuc) gene and GFP (GFP-fLuc) and injected either alone or in combination with human ADSC into the mammary fat pads of recipient nude mice. Tumor formation was monitored based on luciferase expression by high sensitivity binning using an in vivo imaging system. Notably, most mice injected solely with GFP-fLuc MCF-10AT cells exhibited diminishing luminescent signals by day 28 (detailed methods in Supplementary Information), in contrast to the sustained and increasing signals in the ADSC co-injected group (Fig. 2A). In comparison with the MCF-10AT group, mice receiving additive ADSC-injections displayed significantly higher tumor incidence (58% vs. 29%) (Fig. 2B, C) and increased tumor volumes (Fig. 2B, D) at 20 weeks post cell injection. Hematoxylin and Eosin (HE) staining results showed an increased degree of local necrosis and invasion in the co-injected group, as well as enhanced cytological features of malignancy, including cancer cell heterogeneity and the presence of multinucleated and mitotic cells (Fig. 2E). Tumor histopathology was quantified using a semi-quantitative scoring system, which demonstrated a significant increase in malignancy in the ADSC-co-injection group (Fig. 2F). Collectively, our findings suggest that ADSC promotes tumorigenesis and tumor growth of MCF-10AT cells in vivo.

### Grafted rat ADSCs promote tumor formation, progression, and distant metastasis in a DMBA-induced rat breast cancer model

**Fig. 3:**
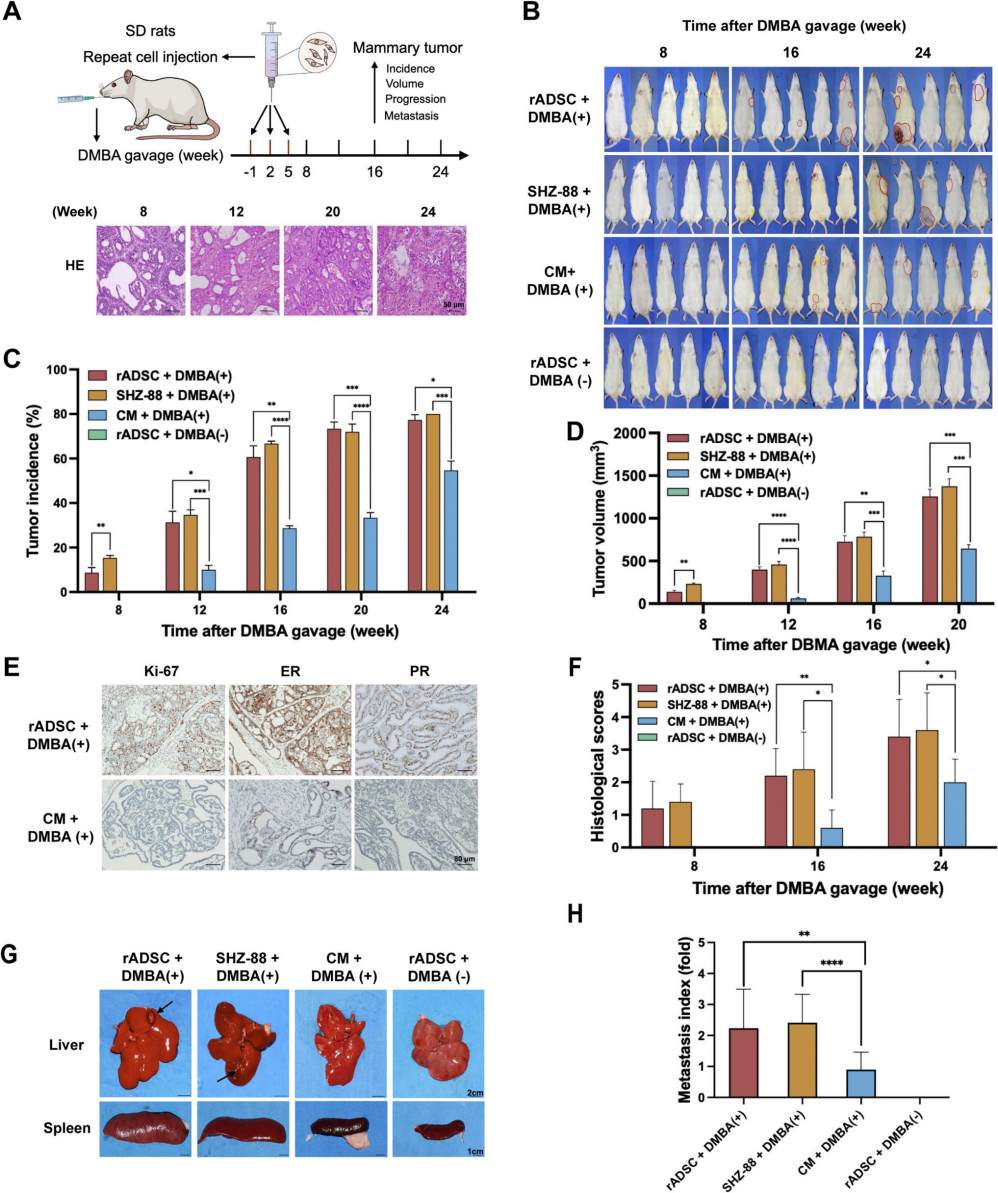
Grafted rat ADSCs (rADSCs) promote tumor formation, progression, and distant organ metastasis in DMBA-induced rat breast cancer models. **A** One week before DMBA induction, rats were randomized and divided into four groups. Three DMBA-induced groups were injected with rADSCs, SHZ-88, or culture medium (CM), and the control group was injected with rADSCs without subsequent DMBA administration. Each rat received three repeated injections at 3-week intervals. Representative HE-stained sections post-DMBA gavage illustrating progression from breast hyperplasia and atypical hyperplasia to carcinoma in situ, then to invasive carcinoma. **B** Representative images of individuals with marked tumors are shown for all DMBA-induced groups at 8, 16, and 24 weeks; no tumors were detected in the control group. **C**, **D** Statistical analyses of tumor incidence (**C**) and tumor volume (**D**) of rADSCs, SHZ-88, CM, and rADSC-DMBA( −) groups at various time points after DMBA induction. **E** IHC results of Ki-67, ER, and PR staining in tumors from the DMBA-induced rADSCs and CM groups. **F** Histological grading scores of tumors from the rADSCs, SHZ-88, CM, and rADSC-DMBA( −) groups at 24 weeks were presented. **G** Photos revealed macroscopic edema and hyperemia in the liver and spleen from rADSCs and SHZ-88 groups with metastatic lesions were indicated by arrows. **H** The tumor metastasis index of all groups at 24 weeks, calculated as the ratio of number of tumor metastases divided by primary tumor weight (in grams). Data in (C, D, F & H) are shown as means ± SD of biological replicates (*n* = 5–8/group at each time point), *p < 0.5, **p < 0.01, ***p < 0.001, ****p < 0.0001

To further investigate the oncogenic effects of grafted ADSCs in a breast cancer-prone environment, we utilized a DMBA-induced breast cancer model in SD rats, with non-treated rats serving as blank controls. Autologous rat ADSCs (rADSCs) were injected subcutaneously into the second mammary gland one week prior to DMBA administration, with three injections repeated at three-week intervals (Fig. 3A, Supplementary Fig. 1B). The SHZ-88 rat breast cancer cells derived from DMBA-induced tumors and culture medium (CM) served as positive and negative controls, respectively. Representative HE-stained sections post-DMBA induction depicted the progression from breast hyperplasia and atypical hyperplasia to carcinoma in situ, leading to invasive carcinoma (Fig. 3A). Interestingly, hyperplasia and atypical hyperplasia occurred earlier in the rADSCs group at week 6, significantly earlier than that in the CM group (week 10), along with an increased rate of local mitosis and tumor invasion. The onset of tumorigenesis, marked by tumors exceeding 3 mm in diameter, was observed as early as 8 weeks post-injection in the rADSCs group, compared to 12 weeks in the CM group. Meanwhile, tumors were more frequently observed in rADSCs-grafted regions of the rat second mammary gland or sub-axillary, suggesting a promoting effect of localized graft on tumor formation (Fig. 3B). Tumor incidence rates were significantly elevated at week-16 in the rADSCs-co-injected group (61%), as high as SHZ-88 group (66%), relative to the CM group (30%) (Fig. 3C). Consistently, the mean tumor volume in the rADSCs group was also significantly increased compared to that in the CM group (Fig. 3D). Moreover, immune-staining signals for Ki-67, estrogen receptor (ER), and progesterone receptor (PR) were more intense in tumors grafted with rADSCs than the CM control (detailed methods in Supplementary Information), indicating a higher malignancy grade in hormone-dependent DMBA-induced mammary tumors (Fig. 3E) [19], which was further validated by histological scoring analysis (Fig. 3F). Notably, no tumors or hyperplasia were observed in untreated rats receiving ADSC transplantation (Fig. 3B, Supplementary Fig. 2A), suggesting that the tumor-promoting effect of ADSC is context-dependent and it is not able to induce primary oncogenesis in normal breast tissues without tumorigenic risk. Importantly, micro-PET-CT imaging combined with gross anatomical observations revealed significant edema, hyperemia, and distant metastatic lesions in the spleen, liver, and other organs for tumors from the rADSCs group (Fig. 3G, Supplementary Fig. 2B, C). The metastasis index, calculated based on the number of metastatic sites relative to the primary tumor weight, was significantly higher in the rADSCs group than controls (Fig. 3H), confirming the in vivo malignancy-promoting effect of ADSC grafting.

### Human ADSCs activate the PI3K–AKT pathway in co-cultured MCF-10AT cells at the gene and protein levels

**Fig. 4:**
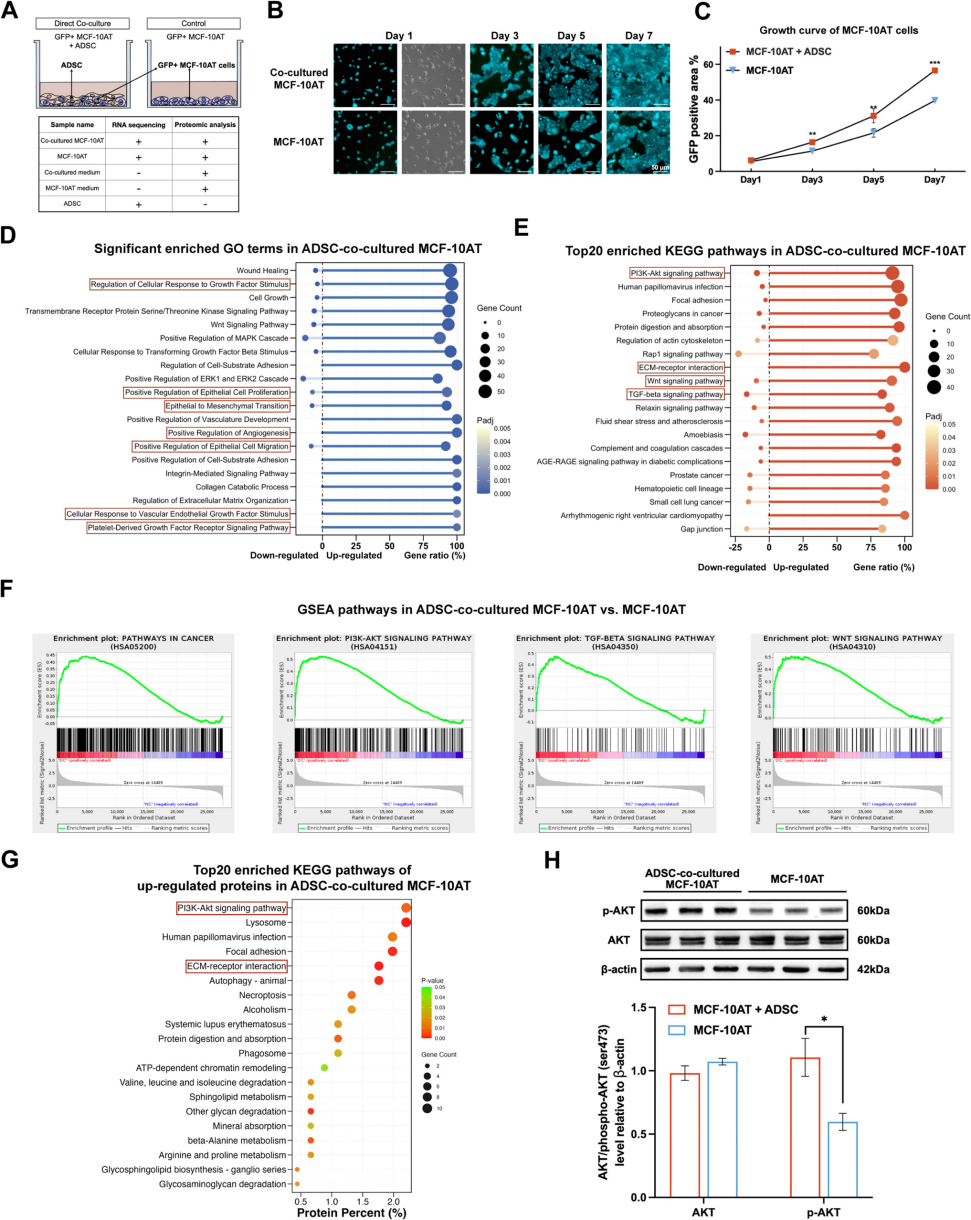
Direct co-culture with human ADSCs activates key cancer-related processes and pathways at the transcriptional level in MCF-10AT cells. **A** Schematic diagram of experiment design. GFP-fLuc MCF-10AT cells were cultured either alone or in direct co-culture with ADSC and sorted for RNA sequencing and proteomic analysis on day 3 and day 7, respectively. Additionally, culture medium from both groups on day 7 was collected for proteomic analysis, and ADSCs were subjected to RNA-seq. **B**, **C** Cell proliferation of ADSC-co-cultured MCF-10AT and mock MCF-10AT cells was captured by fluorescence microscopy (**B**). Statistical analysis of cell growth indicated by GFP aera from day 1 to day 7 is presented (**C**). Data were shown as means ± SD of biological replicates (*n* = 4), **p < 0.01, ***p < 0.001. **D** Gene Ontology (GO) analysis identified 20 significantly enriched biological processes in ADSC-co-cultured MCF-10AT cells. **E** Top20 enriched KEGG pathways in MCF-10AT cells stimulated by ADSC are displayed. **F** Gene Set Enrichment Analysis (GSEA) shows enriched genes in pathways in cancer, PI3K–AKT, TGF-beta, and Wnt signaling pathways in direct co-cultured MCF-10AT cells. **G** KEGG pathway analysis identified top20 pathways enriched by upregulated proteins from co-cultured MCF-10AT cells. **H** Western blot analysis of total AKT, phospho-AKT (Ser473) and β-actin expression in co-cultured and alone cultured MCF-10AT cells. Data are shown as means ± SD of three biological replicates for each group, *p < 0.05

To elucidate the cellular processes of MCF-10AT cells co-existing with ADSCs, GFP-fLuc MCF-10AT cells were cultivated either alone or in direct co-culture with ADSCs (Fig. 4A), a setup more similar to clinical ADSC-assisted fat grafting. Consistent with transwell assay results, direct co-culture significantly increased the proliferation of MCF-10AT cells (Fig. 4B, C). Notably, direct co-culture induced a higher proliferation rate in MCF-10AT cells compared to transwell-mediated indirect co-culture (Supplementary Fig. 3A, B). After a 3-day direct co-culture, GFP-positive MCF-10AT cells were sorted for RNA sequencing. Additionally, MCF-10AT cells and the culture medium after 7-day co-culture were subjected to data-independent acquisition (DIA) proteomic analysis (detailed methods in Supplementary Information). Differential expression analysis identified 908 downregulated and 1537 upregulated genes with fold-change > 2.0 (Supplementary Fig. 4A). Gene Ontology (GO) analysis of dysregulated genes revealed positive regulation of biological processes related to epithelial cell proliferation and migration, angiogenesis, and EMT, and response to growth factor stimuli (Fig. 4D). Kyoto Encyclopedia of Genes and Genomes (KEGG) pathway analysis of differentially expressed genes (DEGs) showed significant enrichment in key oncogenic pathways, such as PI3K–AKT pathway, ECM-receptor interaction, Wnt, and TGF-beta signaling pathways (Fig. 4E), which was further confirmed by Gene Set Enrichment Analysis (GSEA) (Fig. 4F). Intersection analysis of core genes enriched in GSEA pathways revealed 45 genes that overlapped between the PI3K–AKT pathway and pathways in cancer. These highly expressed genes in ADSC-stimulated MCF-10AT cells may represent a unique oncogenic signature, which was defined as the oncogenic PI3K–AKT pathway gene signature (Supplementary Fig. 4B, C).

DIA proteomic analysis identified 728-downregulated and 454-upregulated proteins (differentially expressed proteins, DEPs) in co-cultured MCF-10AT cells compared to mock control (Supplementary Fig. 5A). A positive correlation between dysregulated transcripts and proteins was observed (Pearson’s correlation coefficient = 0.52) (Supplementary Fig. 5B). Consistent with transcriptomic alterations, upregulated proteins upon ADSC co-culture were also involved in the PI3K–AKT pathway and ECM–receptor interaction (Fig. 4G). Correlation analysis between proteins from co-cultured and mock MCF-10AT cells revealed that certain membrane proteins and receptors (ITGB1, IGF1R, etc.) were consistently expressed in MCF-10AT cells at high levels (Supplementary Fig. 5C). Intriguingly, most oncogenic PI3K–AKT pathway signature proteins were remarkably upregulated in co-cultured MCF-10AT cells (Supplementary Fig. 5D). Additionally, western blot analysis (detailed methods in Supplementary Information) revealed elevated phospho-AKT (Ser473) levels in co-cultured MCF-10AT cells without significant changes in total AKT expression, indicating the activation of the PI3K–AKT pathway (Fig. 4H).

### Human ADSC secretome activates the PI3K–AKT signaling pathway in co-cultured MCF-10AT cells

**Fig. 5:**
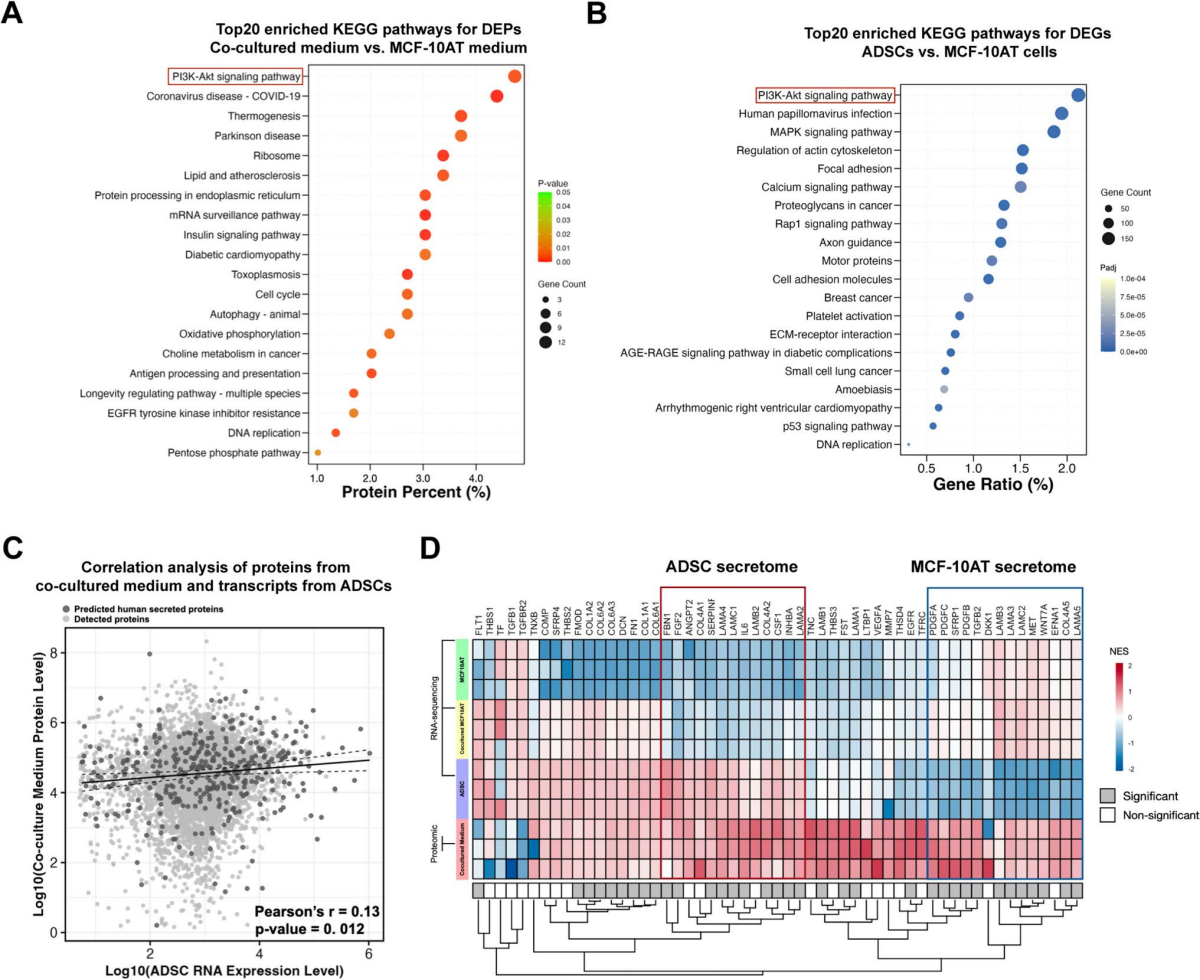
The paracrine effect of human ADSCs activates the oncogenic PI3K–AKT signaling pathway at the protein level in MCF-10AT cells. **A** KEGG analysis for DEPs in ADSC/MCF-10AT co-cultured medium vs. MCF-10AT medium. Top20 enriched pathways are displayed. **B** KEGG analysis identified top20 pathways enriched by DEGs from ADSCs and MCF-10AT cells. **C** Correlation analysis of proteins from co-cultured medium and transcripts from ADSCs are presented (Pearson’s *r* = 0.13, *p* = 0.0119). **D** The heatmap displaying protein expression in the ADSC/MCF-10AT co-cultured medium and gene expression in ADSCs, co-cultured MCF-10AT, and MCF-10AT cells. Proteins from the ADSC secretome are highlighted with a red box, while those from the MCF-10AT secretome are marked with a blue box. Grey and white bars marked significant and non-significant differences in protein expression, respectively

Considering the consistent performance of growth stimulation in MCF-10AT cells in transwell and direct co-culture assays, we postulated that the paracrine proteins secreted by ADSCs may activate the oncogenic PI3K–AKT pathway and further carcinogenesis. To identify these paracrine proteins, the medium after ADSC/MCF-10AT co-culture for 7 days was subjected to proteomic analysis. Further KEGG analysis of DEPs in co-cultured medium revealed significant enrichment in PI3K–AKT pathway (Fig. 5A). Meanwhile, the transcripts from ADSCs were also determined by RNA sequencing analysis for comparison. Coincident with co-cultured MCF-10AT cells (Fig. 4E), PI3K–AKT and ECM-receptor interaction pathways were also enriched in ADSCs relative to mock MCF-10AT cells (Fig. 5B). Correlation analysis between proteins in co-culture medium and transcripts from ADSCs demonstrated that 93% of proteins correlated with mRNA transcripts in ADSCs (Fig. 5C). To identify potential PI3K–AKT activating factors from ADSCs, secreted proteins from proteomic data were correlated with transcripts in ADSCs or co-cultured MCF-10AT cells. The enriched secreted proteins in the co-culture medium that correlated positively with transcripts in ADSCs or co-cultured MCF-10AT cells were identified as ADSC secretome or MCF-10AT secretome, respectively (Fig. 5D). This analysis demonstrated that some proteins in ADSC secretome, identified as upstream regulators of oncogenic PI3K–AKT, MAPK, and JAK/STAT3 pathways, such as FGF2, IL6, and CSF1 [20–22], were highly expressed at the transcriptional level in ADSCs but not in MCF-10AT cells (Fig. 5D). Interestingly, the association between the protein levels of the potential MCF-10AT secretome (TGFB2, WNT7A, PDGFA, PDGFB, etc.) and mRNA levels in MCF-10AT cells was much stronger than that in ADSCs (Fig. 5D). We propose that these growth factors might be released by MCF-10AT cells in an autocrine pathway, which may stimulate or enhance downstream signaling, such as TGF-beta, Wnt, and/or PI3K–AKT pathways to maintain their pre-malignancy [23–25].

To analyze functional interactions between ADSCs and co-cultured MCF-10AT cells, we used the STRING database to integrate proteins from the ADSC secretome with oncogenic PI3K–AKT signature genes and membrane receptors enriched in PI3K–AKT, TGF-beta, and Wnt pathways from co-cultured MCF-10AT cells (Supplementary Fig. 6A–D) (detailed methods in Supplementary Information). We unveiled two pairs of upregulated ligand-receptors: FGFR1-FGF2 and GSF1R-CSF1, which may coordinate the activation of the PI3K–AKT pathway by ADSCs in MCF-10AT cells through cell–cell paracrine interactions (Supplementary Fig. 6A, B). Additionally, we constructed PPI networks of the MCF-10AT secretome to illustrate potential autocrine pathways, which showed high-degree interactions of PDGFs-PDGFRs, WNT7A-FZDs/LRPs, and TGFB2-TGFBRs (Supplementary Fig. 6C, D). In conclusion, our findings indicate that ADSCs enhance the tumorigenic potential of premalignant MCF-10AT cells, possibly through the secretion of growth factors and cytokines that can activate the oncogenic PI3K–AKT and related signaling cascades. Specifically, synergistic interactions of the paracrine ADSC secretome and autocrine MCF-10AT secretome collectively support the proliferation and survival of premalignant cells, thus promoting carcinogenesis.

### Human ADSCs promote cell growth and migration of MCF-10AT cells through the PI3K–AKT pathway

**Fig. 6:**
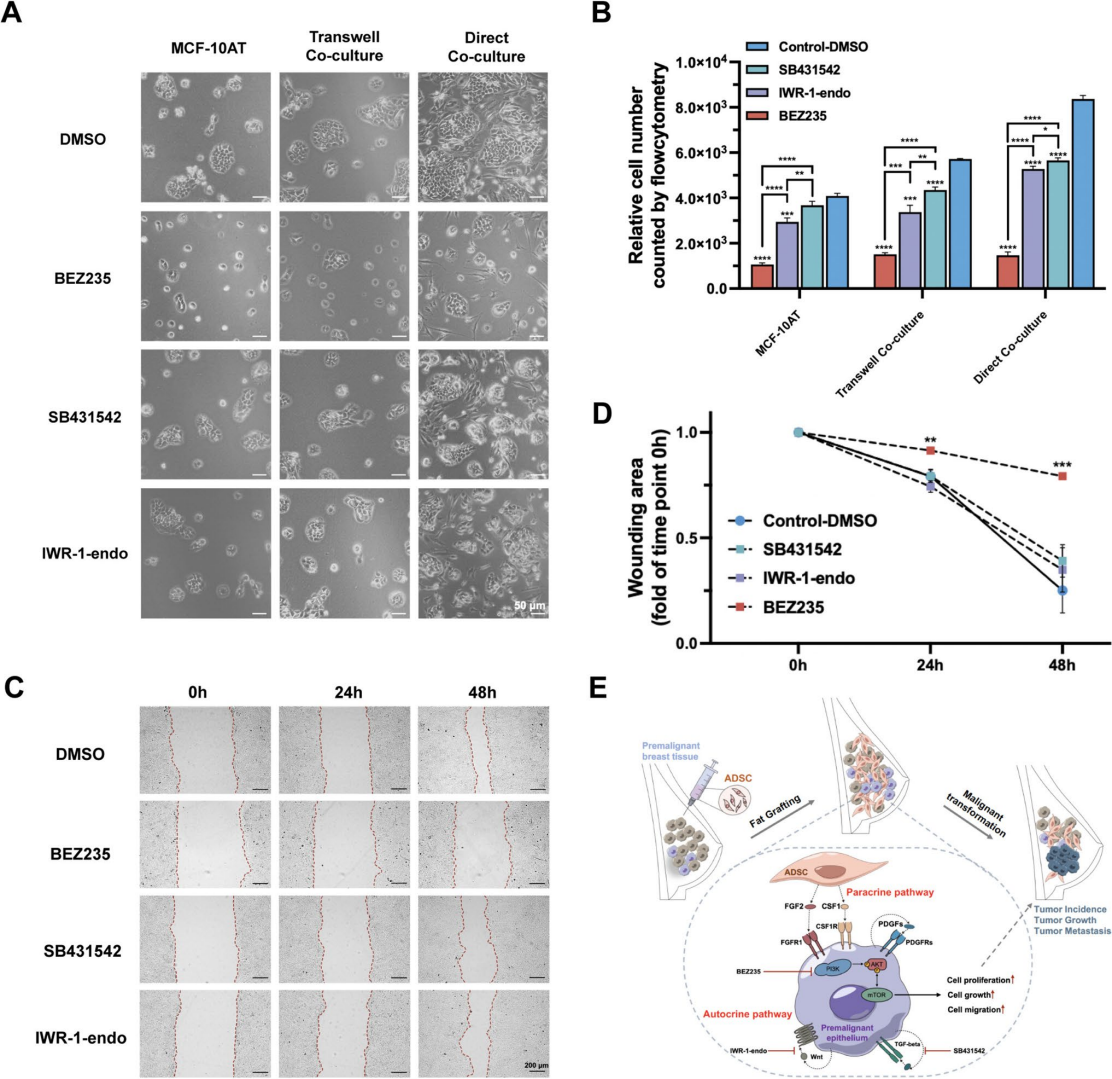
Human ADSCs promote cell growth and migration of MCF-10AT cells through PI3K–AKT pathway. **A**, **B** The effects of PI3K–AKT, TGF-beta, and Wnt pathways on the proliferation of MCF-10AT cells, either cultured alone or co-cultured with ADSCs, were investigated using pathway-targeted inhibitors: BEZ235 (0.5 μM) for PI3K, SB431542 (10 μM) for TGF-beta, and IWR-1-endo (20 μM) for Wnt. The growth-promoting effects of ADSCs were evaluated in both transwell and direct co-culture setups, treated with pathway inhibitors and DMSO solvent. MCF-10AT cells were counted by flow cytometry after three days of culture. Data are shown as means ± SD of biological replicates (*n* = 3), *p < 0.05, **p < 0.01, ***p < 0.001, ****p < 0.0001. **C**, **D** The effects of PI3K–AKT, TGF-beta, and Wnt pathways on the migration of mock or ADSC-co-cultured MCF-10AT cells, were assessed using a transwell wound healing assay. Each group was treated with the corresponding pathway inhibitor or DMSO as a control. Cell mobility was evaluated by measuring the migration area at 24 and 48 h compared to the baseline (0 h) in ImageJ software. Data are shown as means ± SD of biological replicates (*n* = 3), **p < 0.01, ***p < 0.001. **E** The cell–cell interaction model illustrating the potential synergistic effect between ADSC paracrine factors and MCF-10AT auto-activated pathways in enhancing the tumorigenic potential of MCF-10AT cells

Considering the enrichment of PI3K–AKT, TGF-beta, and Wnt signaling pathways in MCF-10AT cells, directly or indirectly activated by ADSCs, we utilized selective inhibitors (BEZ235, a PI3K inhibitor; SB431542, a TGF-beta inhibitor; IWR-1-endo, a Wnt inhibitor) to examine the exact contribution of each pathway to the oncogenic features of MCF-10AT cells. As expected, blocking these three pathways significantly diminished the growth-promoting effect of ADSCs, with the PI3K inhibitor showing the most pronounced effect (Fig. 6A, B). This finding aligns with our hypothesis that the TGF-beta and Wnt inhibitors primarily affect auto-activated signaling pathways in MCF-10AT cells following interaction with ADSCs. Consistently, direct co-culture with ADSCs resulted in a higher proliferation rate than transwell-mediated indirect co-culture in controls (Supplementary Fig. 7A), underscoring the pivotal role of proximal cell–cell contact or higher concentrations of paracrine proteins in enhancing the activity of ADSC-secreted factors. In wound healing assays, the PI3K inhibitor markedly suppressed cell mobility, whereas the other two inhibitors had minimal impact on cell migration (Fig. 6C, D). Collectively, these findings highlighted the predominant role of PI3K–AKT pathway in conferring oncogenic features to MCF-10AT cells induced by ADSCs.

## Discussion

The long-term safety of ADSC-assisted fat transplantation remains controversial, with epidemiological studies presenting positive yet contentious outcomes [15, 16], whereas experimental and mechanistic studies in cancer cell lines and animal models indicate a potential risk for tumorigenesis [26]. Human ADSCs have been reported to promote the proliferation and metastasis of residual tumor cells through secreted CXCLs, IL8, and exosomes containing Wnt ligands [27–29]. Intriguingly, Kucerova et al. demonstrated that the growth-promoting effects of ADSCs could simultaneously enhance the chemosensitivity of breast cancer cells [30]. Considering the low incidence of carcinogenesis in clinical CAL recipients and the heterogeneity of individuals, we propose that these cases might be elicited by the interaction between the genetic or nongenetic background and transplanted adipocytes. In this study, we demonstrated that ADSCs promoted oncogenic changes in vitro and increased tumor incidence in vivo in premalignant breast epithelial MCF-10AT cells (Figs. 1, 2). We also validated the tumor-promoting role of ADSCs in DMBA-induced rat breast cancer, a primary tumor-origin model, while ADSC transplantation alone did not induce tumorigenesis in non-treated rats (Fig. 3). This study unifies the role of ADSCs in breast oncogenesis, demonstrating a context-dependent effect through paracrine oncogenic factors, followed by activation of PI3K–AKT pathways in premalignant lesions.

Human MCF-10AT cells are established by transfecting the *T24 c-Ha-ras* oncogene into benign MCF-10A breast epithelial cells [18] and possess the ability to develop from premalignant hyperplastic changes into heterogenous malignancies, with a mean tumor-forming rate of 25% [31, 32]. Co-injection with ADSCs significantly increased the tumor incidence, volume, and histological grade of MCF-10AT cell-derived tumors, demonstrating the role of ADSCs in promoting tumorigenic potential of premalignancy, such as those with constitutively expressed *c-Ha-ras*, but not in initiating tumors from healthy tissues, as further confirmed in the DMBA-induced model (Figs. 2, 3). Normal mammary ductal epithelial tissues exposed to DMBA undergo a stepwise progression from hyperplasia to atypical hyperplasia, carcinoma in situ, and invasive carcinoma, highly similar to human breast cystic hyperplasia [33]. It is worth noting that the DMBA-induced model primarily develops hormone-positive breast cancer [34], and the role of ADSCs in the formation and progression of triple-negative breast cancer (TNBC) should be further investigated. However, both *c-Ha-ras* in MCF-10AT cells and DMBA in rats may serve as key oncogenic drivers with ADSC acting as a promoter in carcinogenesis. Therefore, identifying intrinsic premalignant or susceptible factors, such as genetic alterations beyond DMBA treatment, will be essential for mitigating the risk associated with ADSC-enriched fat transplantation in the future.

To understand the mechanisms underlying the role of ADSCs in tumorigenesis, we comprehensively analyzed the transcriptomic and proteomic changes in ADSCs, ADSC-co-cultured MCF-10AT cells, and co-cultured medium. The multi-omics analysis revealed marked activation of the PI3K–AKT, TGF-beta, and Wnt signaling pathways in MCF-10AT cells (Figs. 4, 5), with PI3K–AKT emerging as the dominant oncogenic pathways activated in both co-cultured MCF-10AT cells and ADSCs. This suggests that PI3K–AKT pathway may be crucial for MCF-10AT cell oncogenesis via a paracrine pathway and for ADSC growth through an autocrine pathway. Through protein–protein interaction analysis based on ADSC secretome, we identified specific ligand-receptor interaction pairs—FGFR1-FGF2 and CSF1R-CSF1 (Supplementary Fig. 4), which play essential roles in the activation of PI3K–AKT signaling pathway in cancer progression, interaction with cancer-associated fibroblasts, therapy resistance, and epithelial-mesenchymal transition [22, 35–37]. Importantly, blocking PI3K–AKT signaling, a pathway frequently dysregulated across various breast cancer subtypes [38, 39], strikingly diminished the tumor-promoting effect of ADSCs (Fig. 6), underscoring the predominant role of this oncogenic pathway in tumor formation and progression. Co-injection of PI3K inhibitors may help prevent carcinogenesis during ADSC-associated transplantation in clinical applications. In sum, we propose a cell–cell interaction model in which paracrine signaling from ADSCs and autocrine signaling from premalignant cells collectively contribute to tumor formation (Fig. 6E). The auto-activated PDGFs, TGF-beta, and Wnt signaling pathways, which are strictly regulated in normal breast epithelium [23–25], may serve as preconditions for oncogenesis in premalignant lesions.

Despite these findings, several limitations of this study warrant consideration. Although the MCF-10AT cell line and the DMBA-induced rat model effectively reflect certain early tumorigenic events, they do not fully encompass the complexity of human breast carcinogenesis. Utilizing more sophisticated models, such as genetically engineered mouse models that closely replicate the genetic alterations observed in human breast cancer, could provide deeper insights into ADSC-mediated tumor initiation. While our models recapitulate paracrine interactions in premalignant context, they do not fully mirror clinical ADSC-assisted fat grafting where cells are dispersed with in adipose stroma. Future studies using orthotopic fat pad engraftment models may better approximate clinical scenarios. In addition, we observed significant activation of the PI3K–AKT pathway in this tumorigenic process, while the specific upstream factors involved in activating these oncogenic pathways need to be elucidated.

Conclusively, our data demonstrated a synergistic role between ADSC paracrine factors and auto-activated pathways in MCF-10AT cells, promoting tumorigenic potential via activation of the oncogenic PI3K–AKT pathway. This model may help clarify ongoing concerns regarding the risk of ADSC-enriched fat grafting. We also emphasize the importance of pre-clinical screening for breast cancer risk factors, such as atypical dysplasia, before breast reconstruction and augmentation.

## Supplementary Information

Below is the link to the electronic supplementary material.

Supplementary file1 (DOCX 26790 KB)

## Data Availability

The data that support the findings of this research are available from the corresponding author upon reasonable request.
