# Supplementary material for: Adipose-derived stem cells enhance the tumorigenic potential of pre-malignant breast epithelial cells through paracrine activation of PI3K–AKT pathway
Source: Breast Cancer. 2025 Feb 28;32(3):552–65. doi: 10.1007/s12282-025-01686-7 (PMC11993506; doi:10.1007/s12282-025-01686-7)

**Table of Contents**

**Supplementary Methods and Materials**

**Supplementary Tables**

**Supplementary Figures & Legends**

**Source Data**

**Supplementary Methods and Materials**

**Establishment of GFP-fLuc MCF-10AT cells**

To track MCF-10AT cells in both direct co-culture and *in vivo* growth, a lentivirus containing firefly luciferase (fLuc) gene and enhanced green fluorescent protein (GFP) was prepared (Invitrogen, USA). MCF-10AT cells that underwent transduction were selected by G418 (NC9227938, Invitrogen, USA) and maintained in routine culture for subsequent *in vitro* and *in vivo* experiments.

**Detection of GFP-fLuc MCF 10AT cells by *in vivo* bioluminescent imaging**

Following subcutaneous injection of GFP-fLuc MCF-10AT cells, mice were anesthetized for examination with high-sensitivity binning using the *in vivo* imaging system (IVIS^TM^) Living Image software (Xenogen, USA) at different time points. In detail, five minutes prior to imaging, each mouse received an injection of luciferin (Synchem, Germany) at a dose of 3 mg/100 g body weight. General anesthesia was induced using 3% isoﬂurane at a rate of 0.5 L/min and maintained with continuous administration of 1.5% isoflurane with an air flow of 0.3 L/min (R550, RWD Life Science, China). After photographic image acquisition, anterior and posterior luminescent images were taken. The resulting images were automatically superimposed to identify optical signals. Regions of interest were manually drawn to quantify the relative signal intensity during data postprocessing. The optical signal was expressed as photon intensity in units of photons/second (p/s) within the region of interest. In all experiments, mice were allowed to recover for one hour before being returned to group cages.

**Transwell co-culture for cell proliferation and migration assays**

The impact of ADSCs on MCF-10AT proliferation was assessed using cell culture inserts with polyethylene terephthalate (PET) membranes (353095, Corning, USA; 0.4μm). MCF-10AT cells were labeled with 1,1’-dioctadecyl-3,3,3’,3’-tetramethylindocarbocyanine (Dil; D3911, Invitrogen, USA) and seeded in the lower chamber of 24-well plates at a density of 3.0×10^4^ cells per well. Dil-labeled MCF-10AT cells were cultured alone or in co-culture with ADSCs (3.0×10^4^ cells per well) that were seeded in the upper chamber. The methylthiazolyldiphenyl-tetrazolium bromide (MTT; 634843, Millipore, USA) cell growth assay was performed according to the manufacturer’s protocol. Meanwhile, the DiI-labeled MCF-10AT cells in the lower chamber were stained with 4’,6-diamidino-2-phenylindole (DAPI, D1306; Invitrogen, USA) and visualized by confocal laser scanning microscope (CLSM Leica TCS -SP2, Germany) at 24 hours, 3 and 5 days.

The migration potential of MCF-10AT cells was evaluated using a scratch wound healing assay in a 6-well transwell chamber (353090, Corning, USA; 0.4μm). MCF-10AT cells were cultured in the lower chamber until they reached 80-90% confluence. A scratch was then created in the cell layer using a 200µl pipette tip. Next, established MCF-10AT cells were cultured either alone or in transwell co-culture with ADSCs (5.0×10^5^ cells/well) in DMEM medium supplemented with 2% FBS and 1% Penicillin-Streptomycin. The average extent of wound closure was evaluated by measuring the wound area using ImageJ software (Version 1.53), and percent gap closure was determined at, 12, and 24 hours, compared to the initial gap area (0 h).

**Western blotting analysis**

FACS-sorted GFP-positive MCF-10AT cells were lysed using RIPA buffer (PR20001, Proteintech, USA) supplemented with phosphatase inhibitor cocktail (P1082, Beyotime, China) and 1mM PMSF (ST2573, Beyotime, China). Protein samples were measured by bicinchoninic acid (BCA) assay (PA1155, Tiangen, China) according to the manufacturer’s instructions. Loaded protein samples were separated by 10% SDS/PAGE (P0012A, Beyotime, China) and transferred to PDVF membranes (ThermoFisher, USA). The membranes were blocked using fast WB blocking solution (PR20034, Proteintech, USA) for 15 minutes at room temperature, then incubated with primary antibodies against AKT at a 1:1000 dilution (9272, Cell Signaling Technology, USA), phospho-AKT (Ser473) at a 1:10,00 dilution (9271, Cell Signaling Technology, USA), and beta-actin at a 1:50,000 dilution (66009-1-Ig, Proteintech, USA) at 4 °C overnight. Subsequently, the membranes were incubated for one hour with HRP-conjugated anti-rabbit (SA00001-2, Proteintech, USA; 1:5000 dilution) and anti-mouse (SA00001-2, Proteintech, USA; 1:5000 dilution) secondary antibodies at room temperature. The detected proteins were visualized using an ECL chemiluminescence kit (P0018S, Beyotime, China).

**Histology and immunohistochemistry**

Breast tissue specimens, tumors, and metastatic lesions were fixed in 4% formalin and subsequently embedded in paraffin. Representative serial specimen sections (5 μm) were stained with hematoxylin and Eosin (HE) for histological examination. For all groups, histological changes and a score grading of randomly chosen fields were evaluated according to previously published criteria (**Supplementary Table 1**) [2]. For immunohistochemical analysis, tissue sections were incubated overnight at 4°C with the following primary antibodies against estrogen receptor alpha (ER; ab32063, Abcam, UK; 1:200), progesterone receptor (PR; ab101688, Abcam, UK; 1:400), pan-cytokeratin (pan-CK; Kit-0009, Maixin, China), and Ki-67 (ab279653, Abcam, UK; 1:1000). Subsequently, sections were treated with secondary antibodies, developed using diaminobenzidine, and counterstained with hematoxylin to visualize the immunoreactivity.

**RNA sequencing**

Direct co-cultured and alone cultured GFP-fLuc MCF-10AT cells were sorted on day 3 (MoFlo Astrios, Beckman Coulter, USA). Total RNA was extracted from sorted MCF-10AT cells using a total RNA extraction kit (4992856, Tiangen Biotech, China). RNA sequencing (RNA-seq) was conducted using the NovogeneX Plus PE150 platform (Novogene, China). Clean reads for analysis were obtained by filtering out reads with N, adapters, and low quality from the raw data. The DEGseq package in R (version 4.3.1) was utilized for differential expression analysis [3], setting the criteria for differentially expressed genes (DEGs) at fold change > 2.0 and p-adj < 0.05, adjusted by the Benjamini-Hochberg (BH) method.

**Proteomic Analysis**

GFP-fLuc MCF-10AT cells, either cultured alone or in direct co-culture with ADSCs, were sorted on day 7, and corresponding culture medium was collected for protein extraction and quantification as previously described. Subsequent protein processing involved denaturation, reduction, alkylation, and digestion with trypsin using the iST Sample Preparation kit (PreOmics, Germany) following the manufacturer’s protocol. Samples were then lyophilized and reconstituted in 0.1% formic acid for analysis by an Orbitrap Exploris 480 mass spectrometer, coupled to an EASY-nanoLC 1200 system (ThermoFisher, USA). Peptides were separated over a 120-minute gradient in a 25 cm analytical column and analyzed in data-independent acquisition (DIA) mode. Data processing was performed using Spectronaut 18 (Biognosys AG, Switzerland) against the UniProt-Homo sapiens database, with a 1% FDR cutoff for peptides and proteins. Quality control of acquired data was performed on the EasyDIA platform ([https://project.omicsolution.com/EasyDIA/](https://project.omicsolution.com/EasyDIA/?，)). The criteria of differentially expressed proteins (DEPs) were set at fold change > 2 and p-value < 0.01.

**Bioinformatics**

The Kyoto encyclopedia of genes and genomes (KEGG) pathway enrichment analysis and functional gene ontology (GO) annotation were conducted using the clusterProfiler (4.8.3) package in R software. Analysis results with BH-adjusted p-value < 0.05 were considered significant. The GO biological process and KEGG-enriched pathways were visualized using the ggplot2 package (3.4.4) in R. Based on the STRING database ([https://string-db.org](https://string-db.org/)), the PPI networks were constructed and analyzed using the Cytoscape software (version 3.10.1). The “degree” and “betweenness” of each node was identified using the CytoNCA plugin to identify key proteins. Gene Set Enrichment Analysis (GSEA) was used to predict the differentially enriched KEGG pathway by online platform provided by Novogene (<https://magic.novogene.com>). The heatmap and volcano plots were generated by pheatmap (1.0.12) and ggplot2 in R environment. The Venn diagram was generated by using VENNY^2.1^ (<https://csbg.cnb.csic.es/BioinfoGP/venny.html>). The gene lists of human secretome and membrane proteome were obtained from the human protein atlas (<https://www.proteinatlas.org/humanproteome/tissue>) [4].

**Statistical analysis**

All data are presented as mean values ± standard error. Unpaired t test or one-way ANOVA was used when appropriate by using Prism 10 software. p-value <0.05 were regarded as statistically signiﬁcant for all results.

**Supplementary Reference**

1. Zhang TY, Tan PC, Xie Y, Zhang XJ, Zhang PQ, Gao YM, et al. The combination of trehalose and glycerol: an effective and non-toxic recipe for cryopreservation of human adipose-derived stem cells. Stem Cell Res Ther. 2020; 11: 460.

2. Mills SW, Musil KM, Davies JL, Hendrick S, Duncan C, Jackson ML, et al. Prognostic value of histologic grading for feline mammary carcinoma: a retrospective survival analysis. Vet Pathol. 2015; 52: 238-49.

3. Love MI, Huber W, Anders S. Moderated estimation of fold change and dispersion for RNA-seq data with DESeq2. Genome Biol. 2014; 15: 550.

4. Uhlén M, Fagerberg L, Hallström BM, Lindskog C, Oksvold P, Mardinoglu A, et al. Proteomics. Tissue-based map of the human proteome. Science. 2015; 347: 1260419.

**Supplementary Table 1: Criteria for grading of proliferative breast lesions**

| Grade (score) | Classification | Description |
| --- | --- | --- |
| 0 | Simple epithelium | Small ducts  Single layer of luminal epithelium*  No nuclear enlargement  No nucleoli or mitoses |
| 1 | Mild hyperplasia | Small ducts  Two or more layers of epithelial cells*  No significant bridging  Variable nuclear contours |
| 2 | Moderate hyperplasia | Mildly distended ducts  Four or more layers of epithelial cells*  Irregular papillary proliferation  Bridging by non-uniform cells  Irregularly shaped lumens  No solidly filled spaces  Indistinct cell boundaries  Variable nuclear contours  Bland chromatin, small nucleoli |
| 3 | Atypical hyperplasia | Grossly distended ducts  Regular micropapillary configuration  Marked cellular proliferation often forming luminal mass  Some regularity (roundness) of spaces  Some loss of polarity  Cells become monotonous  Tendency to clear cytoplasm with distinct borders  Enlarged, non-round hyperchromatic nuclei  Small nucleoli, occasional mitoses |
| 4 | Carcinoma in situ | Distended ducts filled with uniform cells  Rigid intraluminal bridges forming round spaces  Occasional central necrosis  Distinct cell boundaries  Uniform round, hyperchromatic, enlarged nuclei  Prominent nucleoli, frequent mitoses |
| 5 | Invasive carcinoma | Glandular, squamous, or undifferentiated |

**Supplementary Figures & Legends**

**
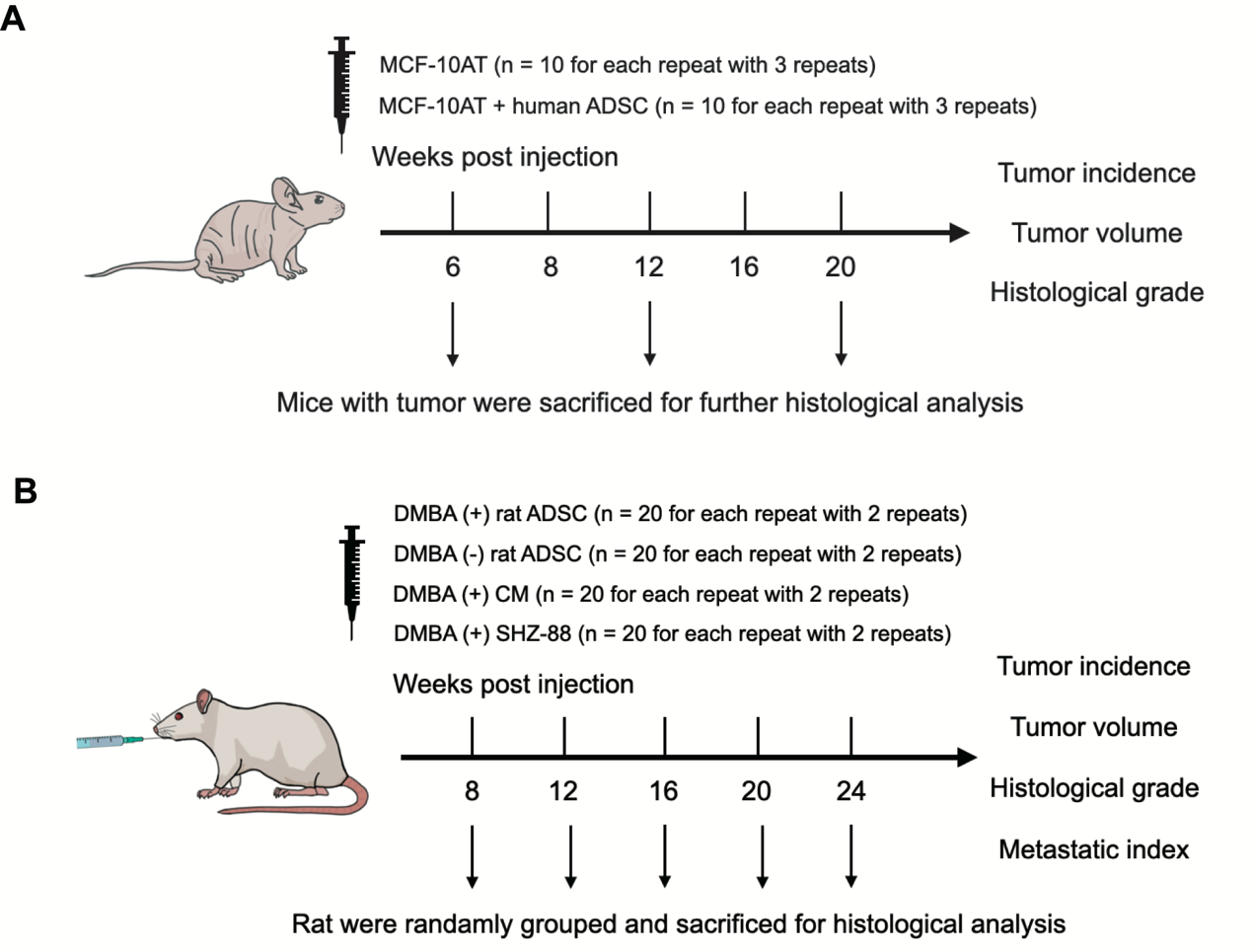
**

**Supplementary Fig. 1: Schematic diagram of the animal experiments.** (A) MCF-10AT xenograft experiments: Two groups of nude mice (n=30 per group) were used across three repeated experiments (n=10 per repeat). Mice bearing tumors were sacrificed at 6, 12, and 20 weeks for histological analysis. (B) DMBA-induced rat experiments: Four groups of rats (n=40 per group) were used in two repeated experiments (n=20 per repeat). In each repeat, 20 rats were randomly divided into five subgroups, and animals were sacrificed at corresponding time points to assess tumor formation and histological evaluation.

**
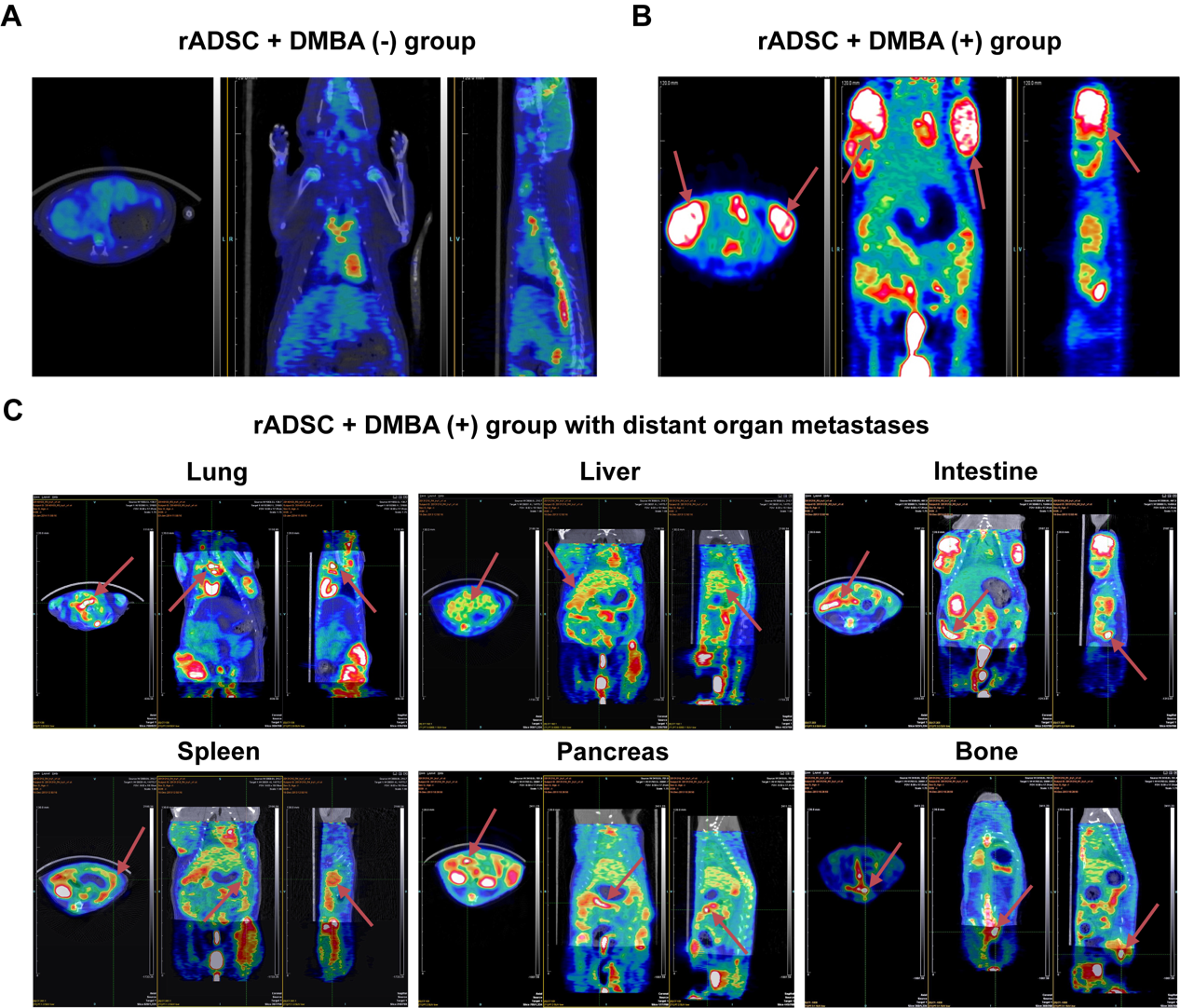
**

**Supplementary Fig. 2: Grafted rADSCs contributed to mammary tumor formation and distant organ metastases in DMBA-induced rats, but not in the non-treated group.** (A, B) Micro-PET-CT results of rats grafted with rADSCs, either subjected to DMBA gavage or not, are displayed. No tumors or hyperplastic breast lesions were detected in healthy rats that received rADSC grafts (A). In rats grafted with rADSCs following DMBA induction, mammary tumors were detected, marked by red arrows (B). (C) Micro-PET-CT results show distant organ metastases in rats that received rADSC injections followed by DMBA gavage, including in the lung, liver, intestine, spleen, pancreas, and bone, as indicated by red arrows. Micro-PET-CT was performed on 5-8 rats per group at each time point, and representative images are shown.

**
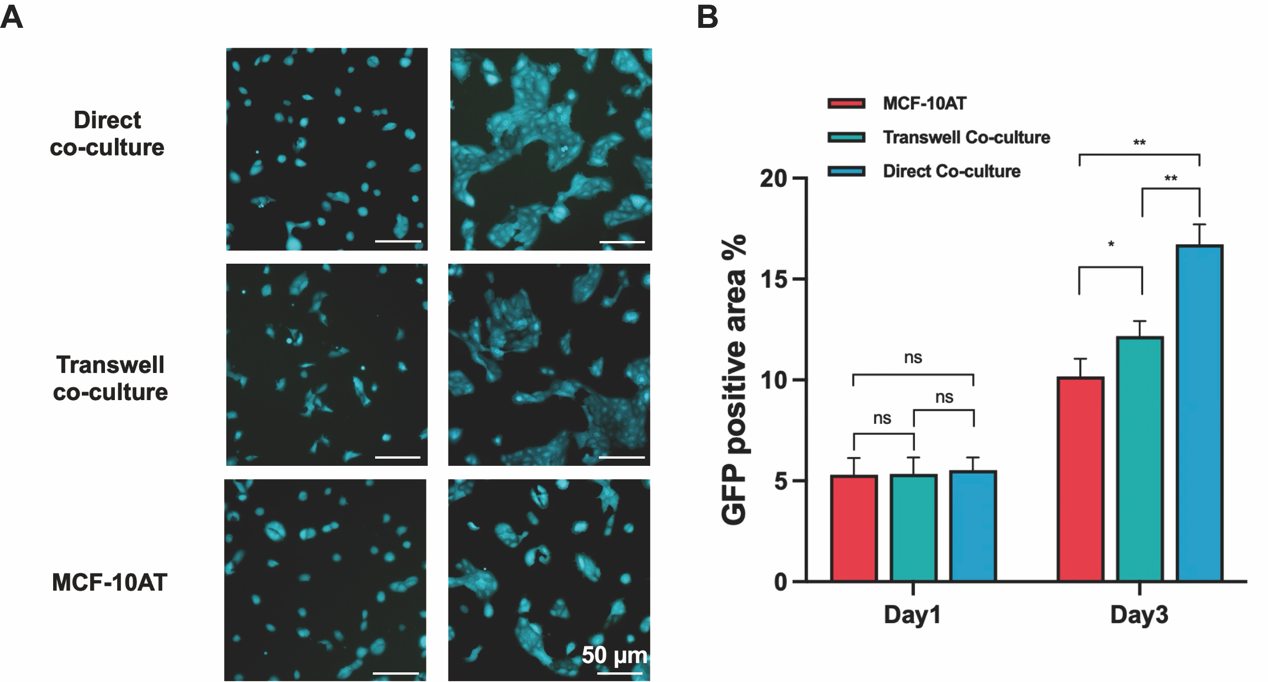
**

**Supplementary Fig. 3:** **Growth-prompting effect of direct and transwell co-culture with ADSCs on MCF-10AT cells.** (A) Cell proliferation of MCF-10AT cells co-cultured with ADSCs (direct or transwell) or mock MCF-10AT cells was captured by fluorescence microscopy. (B) Statistical analysis of GFP-labeled MCF-10AT cell growth, measured as GFP aera at day 1 and day3, is presented. Data were shown as means ± SD of biological replicates (n = 4), ns, not significant, *p < 0.01, **p < 0.001.

**
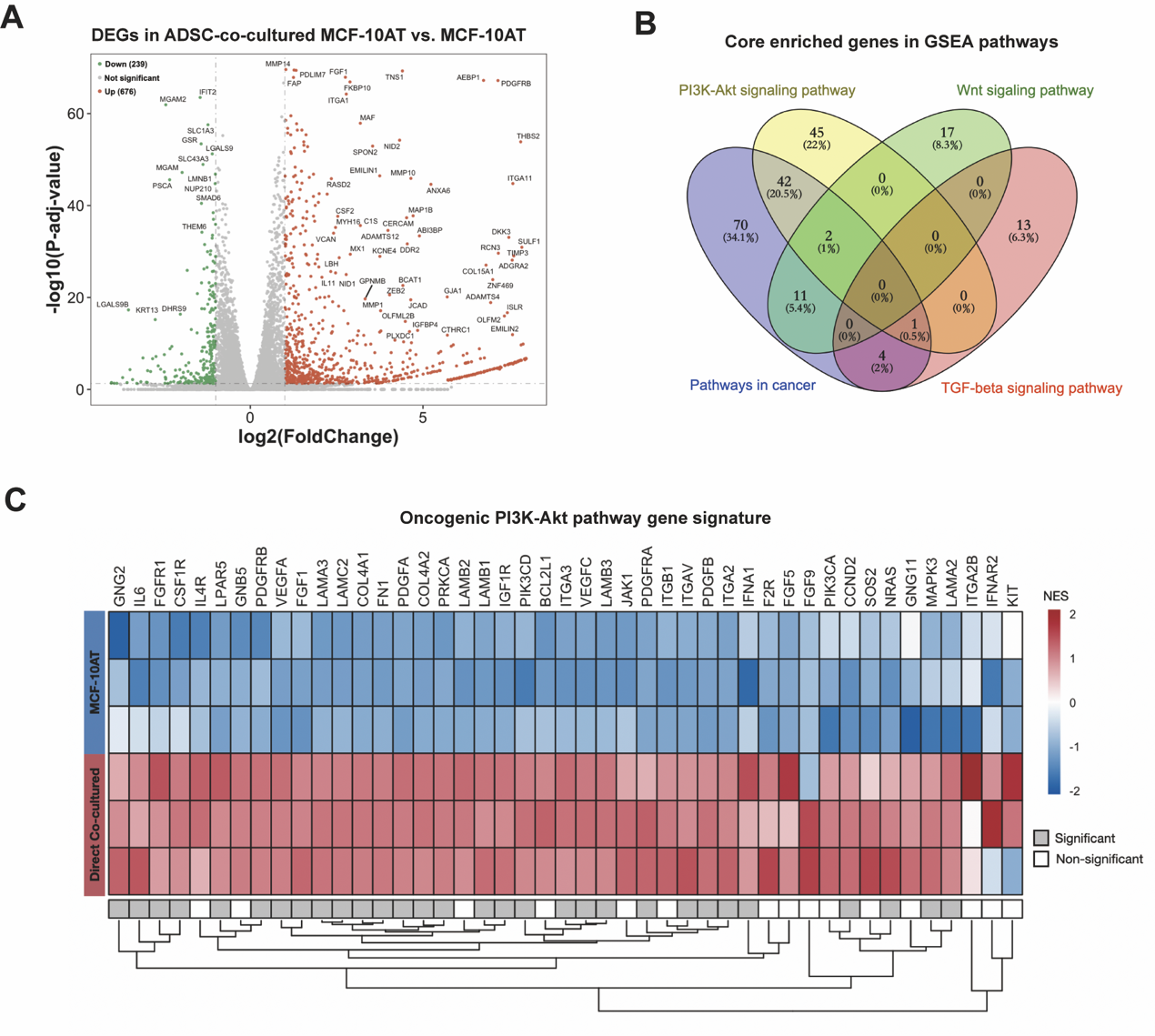
**

**Supplementary Fig. 4: RNA-sequencing analysis of ADSC-co-cultured MCF-10AT cells.** (A) The volcano plot highlighting differentially expressed genes (DEGs) in co-cultured MCF-10AT cells (p-adjust < 0.05, fold change > 2). (B) The Venn diagram shows the core enriched genes in different GSEA pathways. (C) Forty-five genes overlapped between the PI3K-AKT pathway and pathways in cancer were defined as the oncogenic PI3K-KAT pathway gene signature of ADSC-stimulated MCF-10AT cells. Grey and white bars marked significant and non-significant differences in protein expression, respectively.

**
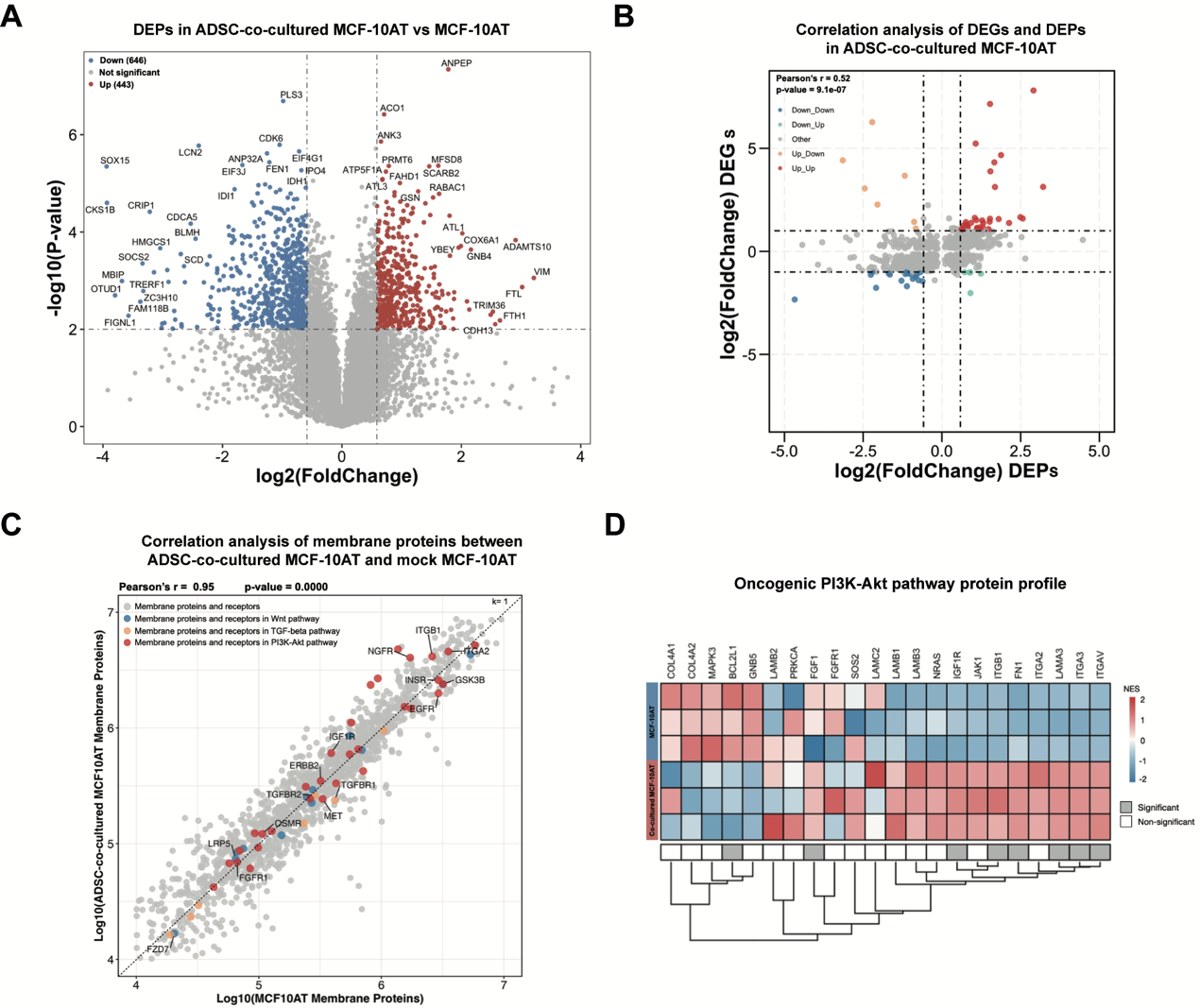
**

**Supplementary Fig. 5: Proteomic analysis of ADSC-co-cultured MCF-10AT cells.** (A) Differentially expressed proteins (DEPs) in co-cultured MCF-10AT cells are highlighted and displayed (p-value < 0.01, fold change > 2). (B) Correlation analysis revealed a moderate positive correlation of the DEGs and DEPs from ADSC-co-cultured MCF-10AT cells with a Pearson's correlation coefficient of 0.52. (C) Correlation analysis of membrane proteins between mock and ADSC-co-cultured MCF-10AT cells (Pearson’s r = 0.95, p < 0.0001). Membrane proteins enriched within PI3K-AKT, TGF-beta, and Wnt signaling pathways are highlighted. (D) The heatmap displays the protein expression levels of oncogenic PI3K-AKT signature genes. Grey and white bars marked significant and non-significant differences in protein expression, respectively.

**
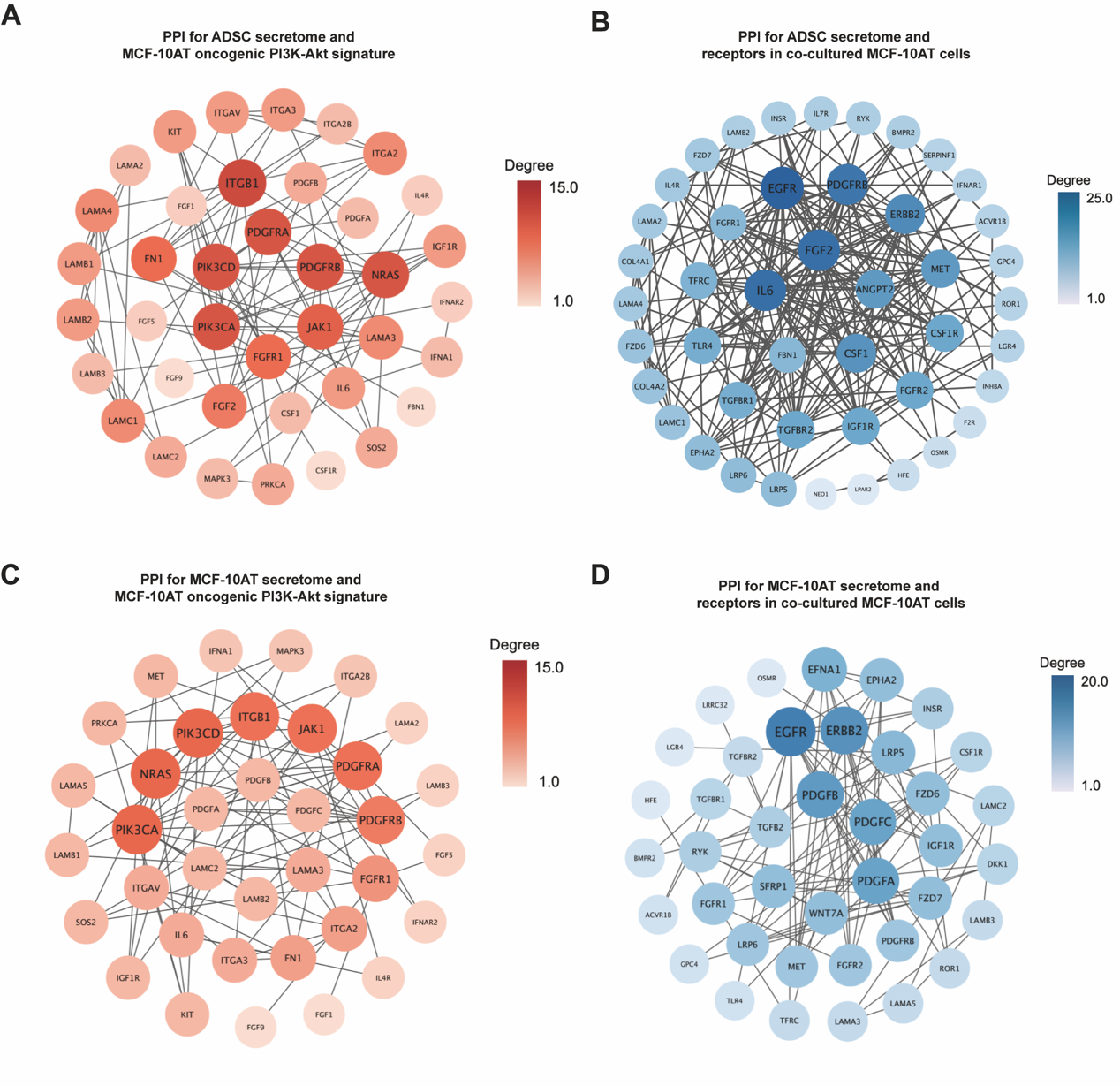
**

**Supplementary Fig. 6: Protein-protein interactions of ADSC and MCF-10AT secretome with oncogenic PI3K-AKT pathways and enriched membrane proteins.** (A, C) The PPI network integrated proteins from the ADSC (A) and MCF-10AT (C) secretome with PI3K-AKT oncogenic signature genes in co-cultured MCF-10AT cells, using STRING database and visualized by Cytoscape. (B, D) The PPI network integrated ADSC (B) and MCF-10AT (D) secretome proteins with membrane receptors enriched in the PI3K-AKT, TGF-beta, and Wnt pathways in co-cultured MCF-10AT cells.

**
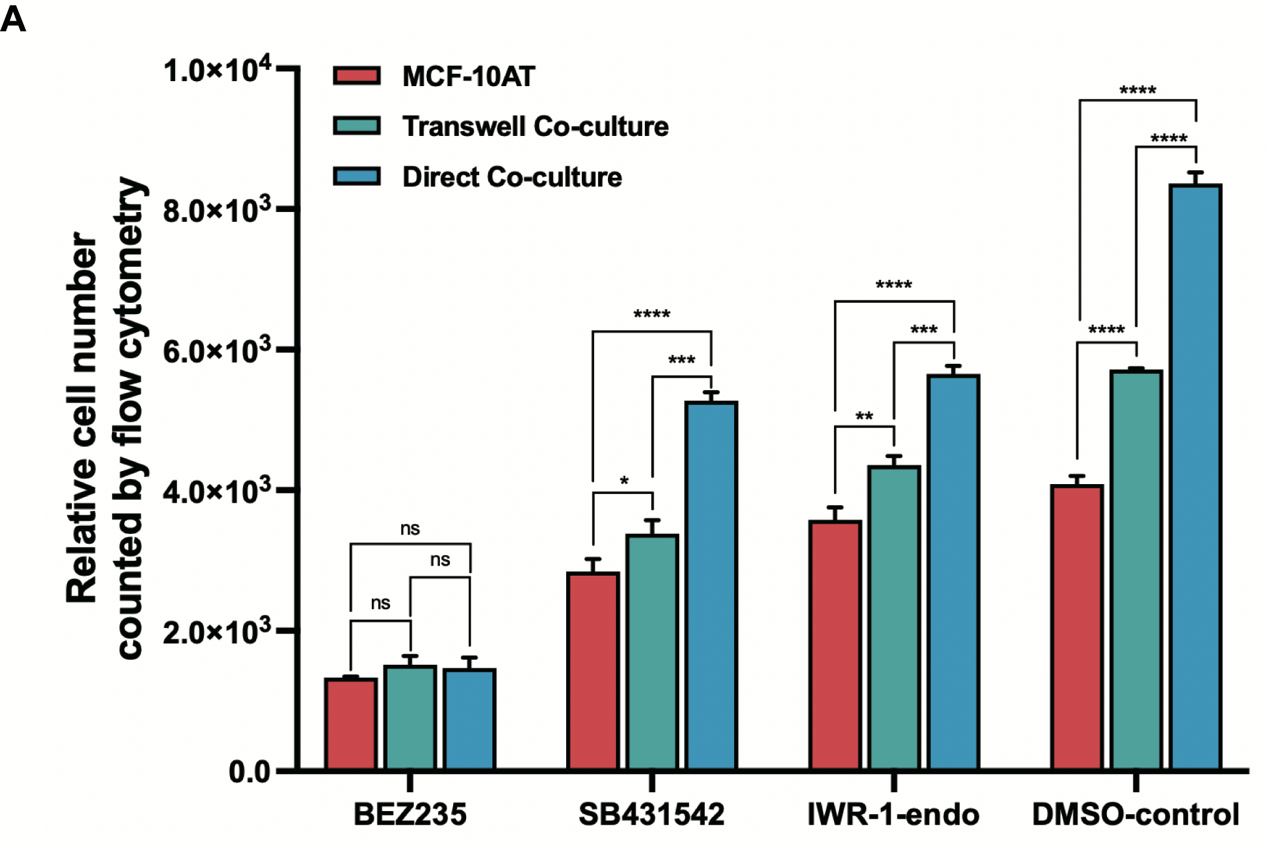
**

**Supplementary Fig. 7: Effect of transwell and direct co-culture with ADSCs on MCF-10AT cell proliferation under different pathway-inhibitor conditions.** (A) MCF-10AT cells (± ADSCs, transwell or direct co-culture) were treated with BEZ235 (0.5 μM), SB431542 (10 μM), IWR-1-endo (20 μM), or DMSO (control) and counted by flow cytometry after three days of culture. Data are shown as means ± SD (n = 3). ns, not significant; *p < 0.05; **p < 0.01; ***p < 0.001; ****p < 0.0001.

**Source Data**


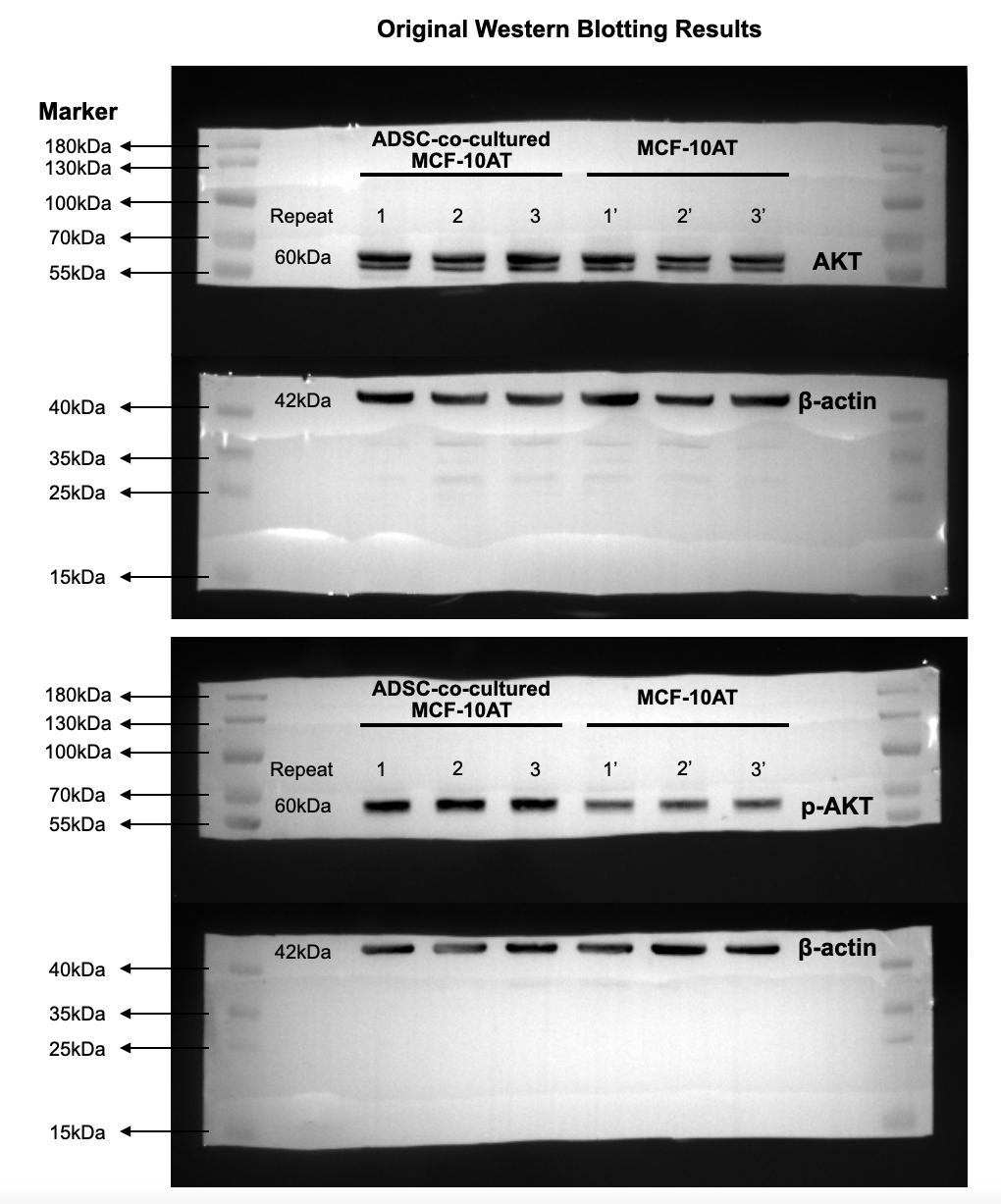

Supplement: Supplementary file 1 — Supplementary file1 (DOCX 26790 KB) [file 12282_2025_1686_MOESM1_ESM.docx]
